# 1,3-Dimethyl-5-(2-methyl­benzyl­idene)pyrimidine-2,4,6(1*H*,3*H*,5*H*)-trione

**DOI:** 10.1107/S1600536809035521

**Published:** 2009-09-09

**Authors:** R. Panchatcharam, V. Dhayalan, A. K. Mohanakrishnan, G. Chakkaravarthi, V. Manivannan

**Affiliations:** a Centre for Research and Development, PRIST University, Vallam, Thanjavur 613 403, Tamil Nadu, India; b Department of Organic Chemistry, University of Madras, Guindy Campus, Chennai 600 025, India; c Department of Physics, CPCL Polytechnic College, Chennai 600 068, India

## Abstract

In the title compound, C_14_H_14_N_2_O_3_, the dihedral angle between the pyrimidine and benzene rings is 14.9 (1)°. The mol­ecular structure is stabilized by weak intra­molecular C—H⋯O inter­actions and the crystal structure exhibits a weak inter­molecular π–π inter­action [centroid–centroid distance = 3.575 (3) Å].

## Related literature

For the biological activity of pyrimidine derivatives, see: Cody *et al.* (1997 ▶); Li *et al.* (1995 ▶). For related structures, see: Da Silva *et al.* (2005 ▶); Rezende *et al.* (2005 ▶). For graph-set notation, see: Bernstein *et al.* (1995 ▶).
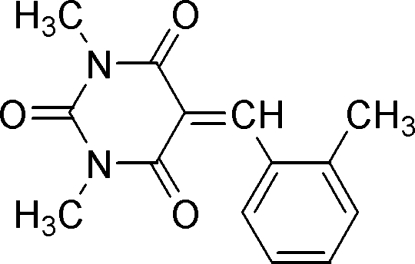


## Experimental

### 

#### Crystal data

C_14_H_14_N_2_O_3_
*M* *_r_* = 258.27
Monoclinic, 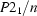

*a* = 8.182 (5) Å
*b* = 8.334 (4) Å
*c* = 18.202 (5) Å
β = 94.267 (5)°
*V* = 1237.7 (10) Å^3^
*Z* = 4
Mo *K*α radiation
μ = 0.10 mm^−1^
*T* = 295 K
0.30 × 0.28 × 0.18 mm

#### Data collection

Bruker Kappa APEXII diffractometer
Absorption correction: multi-scan (*SADABS*; Sheldrick, 1996 ▶) *T*_min_ = 0.971, *T*_max_ = 0.982
16198 measured reflections
3837 independent reflections
2517 reflections with *I* > 2σ(*I*)
*R*_int_ = 0.024

#### Refinement

*R*[*F* ^2^ > 2σ(*F* ^2^)] = 0.072
*wR*(*F* ^2^) = 0.238
*S* = 1.04
3837 reflections
175 parameters
H-atom parameters constrained
Δρ_max_ = 0.52 e Å^−3^
Δρ_min_ = −0.34 e Å^−3^

### 

Data collection: *APEX2* (Bruker, 2004 ▶); cell refinement: *SAINT* (Bruker, 2004 ▶); data reduction: *SAINT*; program(s) used to solve structure: *SHELXS97* (Sheldrick, 2008 ▶); program(s) used to refine structure: *SHELXL97* (Sheldrick, 2008 ▶); molecular graphics: *PLATON* (Spek, 2009 ▶); software used to prepare material for publication: *SHELXL97*.

## Supplementary Material

Crystal structure: contains datablocks global, I. DOI: 10.1107/S1600536809035521/bt5051sup1.cif

Structure factors: contains datablocks I. DOI: 10.1107/S1600536809035521/bt5051Isup2.hkl

Additional supplementary materials:  crystallographic information; 3D view; checkCIF report

## Figures and Tables

**Table 1:** Hydrogen-bond geometry (Å, °)

*D*—H⋯*A*	*D*—H	H⋯*A*	*D*⋯*A*	*D*—H⋯*A*
C2—H2⋯O2	0.93	2.26	2.893 (3)	125

**Fig. 1.**
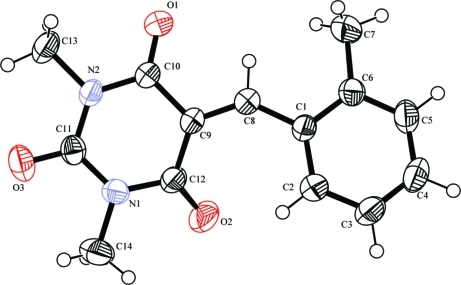
The molecular structure of (I), with atom labels and 30% probability displacement ellipsoids for non-H atoms.

## supplementary crystallographic information

### Comment

Pyrimidine derivatives show biological activities such as antitumor, antibacterial, insulin releasing and anti-inflammatory activities (Cody *et al.*, 1997; Li *et al.*, 1995). The geometric parameters in (I) (Fig. 1) agree with the reported values of similar structures (Da Silva *et al.*, 2005; Rezende *et al.*, 2005).

The dihedral angle between the pyrimidine ring (N1/C11/N2/C10/C9/C12) and benzene ring (C1—C6) is 14.9 (1)°. The molecular structure is stabilized by weak intramolecular C—H···O interactions. The crystal structure exhibits an intermolecular weak π–π interaction[*Cg*1···*Cg*2 = 3.575 (3) Å; symmetry code: -*x*, -*y*, 1 - *z*; *Cg*1 and *Cg*2 are the centroids of N1/C11/N2/C10/C9/C12 and C1—C6 rings, respectively].

The intramolecular C8—H8···O1 and C13—H13B···O3 interactions generate five-membered rings, each with graph-set motif S(5) and C2—H2···O2 interaction generates a seven-membered ring, with graph-set motif S(7) (Bernstein *et al.*, 1995).

### Experimental

To a solution of *o*-tolualdehyde (4.0 g, 33.29 mmol) in dry benzene (80 ml), *N*,*N*-dimethylbarbituric acid (5.72 g, 36.63 mmol), piperidine (0.6 ml) and acetic acid (0.3 ml) were added and refluxed in a RB flask fitted with Dean-Stark apparatus for 12 h. Removal of solvent followed by recrystallizaton from CDCl3 afforded the compound.

### Refinement

H atoms were positioned geometrically and refined using riding model with C—H = 0.93Å and *U*_iso_(H) = 1.2Ueq(C) for C—H and C—H = 0.96Å and *U*_iso_(H) = 1.5Ueq(C) for CH3.

### Crystal data

**Table:**

C_14_H_14_N_2_O_3_	*F*(000) = 544
*M**_r_* = 258.27	*D*_x_ = 1.386 Mg m^−^^3^
Monoclinic, *P*2_1_/*n*	Mo *K*α radiation, λ = 0.71073 Å
Hall symbol: -P 2yn	Cell parameters from 6022 reflections
*a* = 8.182 (5) Å	θ = 2.2–29.8°
*b* = 8.334 (4) Å	µ = 0.10 mm^−^^1^
*c* = 18.202 (5) Å	*T* = 295 K
β = 94.267 (5)°	Block, colourless
*V* = 1237.7 (10) Å^3^	0.30 × 0.28 × 0.18 mm
*Z* = 4	

### Data collection

**Table:**

Bruker Kappa APEXII diffractometer	3837 independent reflections
Radiation source: fine-focus sealed tube	2517 reflections with *I* > 2σ(*I*)
graphite	*R*_int_ = 0.024
ω and φ scans	θ_max_ = 30.7°, θ_min_ = 2.2°
Absorption correction: multi-scan (*SADABS*; Sheldrick, 1996)	*h* = −11→11
*T*_min_ = 0.971, *T*_max_ = 0.982	*k* = −8→11
16198 measured reflections	*l* = −26→26

### Refinement

**Table:**

Refinement on *F*^2^	Primary atom site location: structure-invariant direct methods
Least-squares matrix: full	Secondary atom site location: difference Fourier map
*R*[*F*^2^ > 2σ(*F*^2^)] = 0.072	Hydrogen site location: inferred from neighbouring sites
*wR*(*F*^2^) = 0.238	H-atom parameters constrained
*S* = 1.04	*w* = 1/[σ^2^(*F*_o_^2^) + (0.1214*P*)^2^ + 0.5641*P*] where *P* = (*F*_o_^2^ + 2*F*_c_^2^)/3
3837 reflections	(Δ/σ)_max_ < 0.001
175 parameters	Δρ_max_ = 0.52 e Å^−^^3^
0 restraints	Δρ_min_ = −0.34 e Å^−^^3^

### Fractional atomic coordinates and isotropic or equivalent isotropic displacement parameters (Å^2^)

**Table:**

	*x*	*y*	*z*	*U*_iso_*/*U*_eq_	
C1	0.0138 (2)	0.2553 (2)	0.49118 (10)	0.0391 (4)	
C2	−0.1183 (3)	0.2104 (3)	0.44177 (12)	0.0547 (6)	
H2	−0.1974	0.1406	0.4573	0.066*	
C3	−0.1326 (3)	0.2681 (4)	0.37060 (12)	0.0589 (6)	
H3	−0.2205	0.2370	0.3384	0.071*	
C4	−0.0168 (3)	0.3713 (3)	0.34746 (12)	0.0548 (6)	
H4	−0.0285	0.4147	0.3003	0.066*	
C5	0.1166 (3)	0.4106 (3)	0.39422 (12)	0.0519 (5)	
H5	0.1961	0.4785	0.3775	0.062*	
C6	0.1363 (2)	0.3521 (3)	0.46527 (11)	0.0424 (5)	
C7	0.2861 (3)	0.4007 (4)	0.51198 (15)	0.0714 (8)	
H7A	0.3586	0.3105	0.5188	0.107*	
H7B	0.2552	0.4370	0.5590	0.107*	
H7C	0.3408	0.4859	0.4881	0.107*	
C8	0.0343 (3)	0.2024 (3)	0.56714 (11)	0.0447 (5)	
H8	0.1412	0.2161	0.5873	0.054*	
C9	−0.0641 (2)	0.1379 (2)	0.61602 (10)	0.0390 (4)	
C10	0.0217 (3)	0.0920 (3)	0.68754 (10)	0.0429 (5)	
C11	−0.2160 (2)	−0.0676 (2)	0.71455 (10)	0.0396 (4)	
C12	−0.2395 (3)	0.1068 (3)	0.60570 (11)	0.0449 (5)	
C13	0.0281 (3)	−0.0647 (3)	0.79980 (12)	0.0584 (6)	
H13A	0.1247	−0.1223	0.7885	0.088*	
H13B	−0.0416	−0.1340	0.8257	0.088*	
H13C	0.0589	0.0263	0.8300	0.088*	
C14	−0.4706 (3)	−0.0581 (4)	0.63701 (16)	0.0715 (8)	
H14A	−0.4822	−0.1675	0.6524	0.107*	
H14B	−0.5004	−0.0496	0.5851	0.107*	
H14C	−0.5409	0.0093	0.6636	0.107*	
N1	−0.3007 (2)	−0.0073 (2)	0.65217 (9)	0.0433 (4)	
N2	−0.0600 (2)	−0.0100 (2)	0.73120 (8)	0.0397 (4)	
O1	0.1595 (2)	0.1369 (3)	0.70726 (9)	0.0682 (6)	
O2	−0.3317 (2)	0.1744 (3)	0.56044 (11)	0.0748 (6)	
O3	−0.2774 (2)	−0.1642 (2)	0.75393 (10)	0.0587 (5)	

### Atomic displacement parameters (Å^2^)

**Table:**

	*U*^11^	*U*^22^	*U*^33^	*U*^12^	*U*^13^	*U*^23^
C1	0.0383 (9)	0.0465 (10)	0.0326 (8)	0.0012 (8)	0.0033 (7)	0.0046 (7)
C2	0.0453 (11)	0.0775 (16)	0.0407 (10)	−0.0112 (11)	−0.0004 (9)	0.0047 (11)
C3	0.0505 (12)	0.0883 (18)	0.0367 (10)	0.0003 (12)	−0.0041 (9)	0.0010 (11)
C4	0.0654 (14)	0.0672 (15)	0.0323 (9)	0.0100 (11)	0.0064 (9)	0.0082 (9)
C5	0.0614 (13)	0.0556 (13)	0.0403 (10)	−0.0084 (10)	0.0144 (9)	0.0035 (9)
C6	0.0407 (10)	0.0498 (11)	0.0371 (9)	−0.0012 (8)	0.0061 (7)	−0.0031 (8)
C7	0.0539 (14)	0.104 (2)	0.0549 (14)	−0.0309 (15)	−0.0017 (11)	0.0038 (14)
C8	0.0417 (10)	0.0534 (12)	0.0382 (9)	−0.0060 (9)	−0.0016 (8)	0.0071 (9)
C9	0.0413 (10)	0.0433 (10)	0.0320 (8)	−0.0013 (8)	−0.0009 (7)	0.0049 (7)
C10	0.0415 (10)	0.0535 (11)	0.0330 (9)	−0.0047 (9)	−0.0017 (7)	0.0061 (8)
C11	0.0460 (10)	0.0390 (10)	0.0350 (9)	−0.0009 (8)	0.0100 (7)	−0.0014 (7)
C12	0.0391 (10)	0.0608 (13)	0.0345 (9)	0.0025 (9)	0.0008 (7)	0.0053 (9)
C13	0.0598 (14)	0.0761 (16)	0.0384 (10)	0.0067 (12)	−0.0022 (9)	0.0186 (11)
C14	0.0449 (13)	0.108 (2)	0.0611 (15)	−0.0255 (14)	0.0006 (11)	−0.0008 (15)
N1	0.0378 (8)	0.0532 (10)	0.0387 (8)	−0.0071 (7)	0.0025 (6)	−0.0027 (7)
N2	0.0408 (8)	0.0466 (9)	0.0315 (7)	0.0021 (7)	0.0020 (6)	0.0070 (6)
O1	0.0512 (9)	0.1035 (15)	0.0473 (9)	−0.0257 (9)	−0.0139 (7)	0.0199 (9)
O2	0.0443 (9)	0.1221 (17)	0.0570 (10)	0.0125 (10)	−0.0022 (8)	0.0328 (11)
O3	0.0673 (11)	0.0574 (10)	0.0536 (9)	−0.0123 (8)	0.0186 (8)	0.0089 (7)

### Geometric parameters (Å, °)

**Table:**

C1—C6	1.396 (3)	C9—C12	1.457 (3)
C1—C2	1.404 (3)	C9—C10	1.482 (3)
C1—C8	1.449 (3)	C10—O1	1.217 (3)
C2—C3	1.379 (3)	C10—N2	1.371 (3)
C2—H2	0.9300	C11—O3	1.212 (2)
C3—C4	1.369 (4)	C11—N2	1.376 (3)
C3—H3	0.9300	C11—N1	1.380 (3)
C4—C5	1.373 (4)	C12—O2	1.214 (3)
C4—H4	0.9300	C12—N1	1.391 (3)
C5—C6	1.380 (3)	C13—N2	1.467 (3)
C5—H5	0.9300	C13—H13A	0.9600
C6—C7	1.495 (3)	C13—H13B	0.9600
C7—H7A	0.9600	C13—H13C	0.9600
C7—H7B	0.9600	C14—N1	1.460 (3)
C7—H7C	0.9600	C14—H14A	0.9600
C8—C9	1.355 (3)	C14—H14B	0.9600
C8—H8	0.9300	C14—H14C	0.9600
			
C6—C1—C2	118.38 (18)	C12—C9—C10	117.69 (17)
C6—C1—C8	117.61 (18)	O1—C10—N2	120.04 (18)
C2—C1—C8	123.97 (19)	O1—C10—C9	123.19 (19)
C3—C2—C1	121.1 (2)	N2—C10—C9	116.74 (18)
C3—C2—H2	119.5	O3—C11—N2	121.34 (19)
C1—C2—H2	119.5	O3—C11—N1	121.6 (2)
C4—C3—C2	119.7 (2)	N2—C11—N1	117.08 (17)
C4—C3—H3	120.1	O2—C12—N1	119.8 (2)
C2—C3—H3	120.1	O2—C12—C9	124.2 (2)
C3—C4—C5	119.7 (2)	N1—C12—C9	116.06 (17)
C3—C4—H4	120.1	N2—C13—H13A	109.5
C5—C4—H4	120.1	N2—C13—H13B	109.5
C4—C5—C6	121.9 (2)	H13A—C13—H13B	109.5
C4—C5—H5	119.0	N2—C13—H13C	109.5
C6—C5—H5	119.0	H13A—C13—H13C	109.5
C5—C6—C1	118.95 (19)	H13B—C13—H13C	109.5
C5—C6—C7	118.1 (2)	N1—C14—H14A	109.5
C1—C6—C7	122.9 (2)	N1—C14—H14B	109.5
C6—C7—H7A	109.5	H14A—C14—H14B	109.5
C6—C7—H7B	109.5	N1—C14—H14C	109.5
H7A—C7—H7B	109.5	H14A—C14—H14C	109.5
C6—C7—H7C	109.5	H14B—C14—H14C	109.5
H7A—C7—H7C	109.5	C11—N1—C12	124.58 (17)
H7B—C7—H7C	109.5	C11—N1—C14	117.58 (19)
C9—C8—C1	135.66 (19)	C12—N1—C14	117.7 (2)
C9—C8—H8	112.2	C10—N2—C11	124.92 (16)
C1—C8—H8	112.2	C10—N2—C13	117.15 (18)
C8—C9—C12	127.86 (18)	C11—N2—C13	117.91 (18)
C8—C9—C10	114.43 (18)		
			
C6—C1—C2—C3	−4.0 (4)	C10—C9—C12—O2	159.2 (2)
C8—C1—C2—C3	178.4 (2)	C8—C9—C12—N1	158.5 (2)
C1—C2—C3—C4	−0.2 (4)	C10—C9—C12—N1	−20.2 (3)
C2—C3—C4—C5	3.1 (4)	O3—C11—N1—C12	178.2 (2)
C3—C4—C5—C6	−1.7 (4)	N2—C11—N1—C12	−0.2 (3)
C4—C5—C6—C1	−2.5 (4)	O3—C11—N1—C14	2.9 (3)
C4—C5—C6—C7	179.6 (3)	N2—C11—N1—C14	−175.4 (2)
C2—C1—C6—C5	5.2 (3)	O2—C12—N1—C11	−166.3 (2)
C8—C1—C6—C5	−177.0 (2)	C9—C12—N1—C11	13.1 (3)
C2—C1—C6—C7	−177.0 (3)	O2—C12—N1—C14	8.9 (4)
C8—C1—C6—C7	0.7 (3)	C9—C12—N1—C14	−171.7 (2)
C6—C1—C8—C9	164.5 (3)	O1—C10—N2—C11	179.7 (2)
C2—C1—C8—C9	−17.9 (4)	C9—C10—N2—C11	−2.3 (3)
C1—C8—C9—C12	−3.4 (4)	O1—C10—N2—C13	−2.0 (3)
C1—C8—C9—C10	175.4 (2)	C9—C10—N2—C13	176.01 (19)
C8—C9—C10—O1	14.5 (3)	O3—C11—N2—C10	176.1 (2)
C12—C9—C10—O1	−166.7 (2)	N1—C11—N2—C10	−5.6 (3)
C8—C9—C10—N2	−163.5 (2)	O3—C11—N2—C13	−2.2 (3)
C12—C9—C10—N2	15.4 (3)	N1—C11—N2—C13	176.12 (18)
C8—C9—C12—O2	−22.1 (4)		

### Hydrogen-bond geometry (Å, °)

**Table:**

*D*—H···*A*	*D*—H	H···*A*	*D*···*A*	*D*—H···*A*
C2—H2···O2	0.93	2.26	2.893 (3)	125
C8—H8···O1	0.93	2.28	2.732 (3)	110
C13—H13B···O3	0.96	2.26	2.706 (4)	107
